# Diet-derived transmission of MicroRNAs from host plant into honey bee Midgut

**DOI:** 10.1186/s12864-021-07916-4

**Published:** 2021-08-03

**Authors:** Leila Gharehdaghi, Mohammad Reza Bakhtiarizadeh, Kang He, Taher Harkinezhad, Gholamhosein Tahmasbi, Fei Li

**Affiliations:** 1 Department of Animal Science, Faculty of Agriculture, University of Zanjan, Zanjan, Iran; 2 Department of Animal and Poultry Science, College of Aburaihan, University of Tehran, Tehran, Iran; 3 Ministry of Agriculture and Rural Affairs Key Lab of Molecular Biology of Crop Pathogens and Insects/Institute of Insect Sciences, Zhejiang University, 866 Yuhangtang Road, Hangzhou, 310058 China; 4 Department of Honeybee, Agricultural Research, Education and Extension Organization (AREEO), Animal Science Research Institute of Iran, Karaj, Iran

**Keywords:** miRNA, Cross species, *Apis mellifera*, *Helianthus annuus*, *Ziziphus spina-christi*

## Abstract

**Background:**

MicroRNA (miRNA) is a class of small noncoding RNAs, which targets on thousands of mRNA and thus plays important roles in many biological processes. It has been reported that miRNA has cross-species regulation functions between parasitoid-host, or plant-animal, etc. For example, several plant miRNAs enter into the honey bees and regulate gene expression. However, whether cross-species regulation function of miRNAs is a universal mechanism remains a debate question.

**Results:**

We have evaluated transmission of miRNAs from sunflower and sedr plants into the midgut of honey bee using RNA-Seq analyses complemented with confirmation by RT-qPCR. The results showed that at least 11 plant miRNAs were found in the midgut of honey bee feeding by sunflower and sedr pollen. Among which, nine miRNAs, including miR-30d, miR-143, miR-148a, miR-21, let-7 g, miR-26a, miR-126, miR-27a, and miR-203, were shared between the sunflower- and sedr-fed honey bees, suggesting they might have essential roles in plant-insect interactions. Moreover, existence of these co-shared miRNAs presents a strong evidence to support the successful transmission of miRNAs into the midgut of the insect. In total, 121 honeybee mRNAs were predicted to be the target of these 11 plant-derived miRNAs. Interestingly, a sedr-derived miRNA, miR-206, targets on 53 honeybee genes. Kyoto Encyclopedia of Genes and Genome (KEGG) analyses showed that these target genes are significantly involved in hippo signaling pathway-fly, *Wnt* signaling pathway, and N-Glycan biosynthesis.

**Conclusions:**

In summary, these results provide evidence of cross-species regulation function of miRNA between honeybee and flowering host plants, extending our understanding of the molecular interactions between plants and animals.

**Supplementary Information:**

The online version contains supplementary material available at 10.1186/s12864-021-07916-4.

## Background

MicroRNAs (miRNAs) are a class of non-coding small RNAs (sRNAs), which are functionality significant and approximately 19–24 nucleotide (nt) in size. They are known as regulators of gene expression by binding to open reading frames (ORF) or untranslated region (UTR) of specific mRNAs, targeting them for cleavage or directing translation inhibition at the mRNA level [1]. It has been demonstrated that around 60% of protein coding genes are targets of miRNAs and modulated by these small RNAs [2]. The miRNAs play crucial roles in a wide range of biological processes i.e. developmental timing, cell differentiation, proliferation, apoptosis as well as regulation of a number of important agronomic traits [3–7].

Recent studies revealed that miRNAs have exogenous roles as well. Plant miRNAs are 2′-O-methylated at 3′ end to enhance their stability for the survival in various exogenous environments [8]. By several exogenous paths of intracellular and intercellular transport via P bodies (cytoplasmic bodies), multi vesicular bodies (MVBs) and endosomes [9, 10], plant miRNAs were transported into animals. The plant exogenous miRNAs find their complementary targets and modulate the transcriptional or post-transcriptional processes in animals. Cross species interactions formed by plant miRNA and host mRNA might result into genetic regulation alterations in the host [11]. Up to now, cross-species translocation of diet-derived small RNAs to ingesting organisms has been described in both vertebrates [12] and invertebrates [13, 14].

It has been confirmed for the first time that plant-derived miR168a could be absorbed through the gastrointestinal tract into mammalian liver cells where it inhibits the expression of the human/mouse low-density lipoprotein receptor adapter protein1 (LDLRAP1), and consequently decreases LDL removal from mouse plasma [12]. Consequently, it has been demonstrated that honeysuckle-encoded miRNAs, miR2911 could be taken up via gastrointestinal tract Influenza A virus (IAV)-infected mice and counteract viral infections [15]. As for the arthropods, a number of insects can ingest sRNAs which subsequently regulate the expression of their genes, thus reshaping the phenotype of the animal [16, 17]. In this regard, Zhu et al. reported that plant miRNAs in larval food regulate honey bee caste development [18]. It has been shown that plant miRNAs, which are more frequent in beebread than in royal jelly, can knock out amTOR gene. This gene is linked to cast determination and decrease body and ovary size in honey bee and trigger embryo–larva transition of honeybee worker.

However, systemic uptake of orally ingested foreign miRNAs is negligible and significantly below the levels that required to be biologically relevant when acting through canonical sequence-specific miRNA-mediated mechanisms [19–21]. Hence, the hypothesis of cross-kingdom regulations mediated by exogenous plant miRNAs is still controversial and remains to be clarified. As one of the well-studied pollinators, honey bee provides an excellent model for exploring the role of dietary transfer of small RNAs in communication between plants and pollinators. Though biologically relevant plant-derived miRNAs were not observed in the honey bee, further analysis showed that a highly-conserved plant miRNA, miR156a, was significantly increased in the midgut of honey bee [19]. However, there is still not direct evidence for the transmission of biologically relevant miRNAs to proximal or distal tissues of honey bees [22].

Here, we investigate the transmission of plant miRNAs from two different plant species i.e. sunflower (Order: Asterales, Family: Asteraceae) and sedr (Order: Rosales, Family: Rhamnaceae) into the midgut of honey bees under controlled environmental conditions. To detect mono-floral diet-derived miRNAs in honey bee, we chose sunflower and sedr which were the dominant vegetation in the study region in Iran and had different flowering times than the other plants. The individual honey bees are fed by either sunflower (*Helianthus annuus)* or sedr (*Ziziphus spina-christi)* pollen. Then, the total miRNAs of the midgut tissue of nurse honey bees are sequenced using RNA-Seq technology. The results show that 11 plant miRNAs are found in the midgut of nurse honey bees fed with sunflower pollen or sedr pollen. Nine out of 11 miRNAs are shared between the two groups of adult honey bees, suggesting that these miRNAs have specific features that increase their ability to transmit from the host plant to the honey bee. These data extend our understanding of cross species regulation of plant-derived miRNAs in animals.

## Results

### RNA-seq of sRNA libraries from sunflower and Sedr pollen

**Fig. 1:**
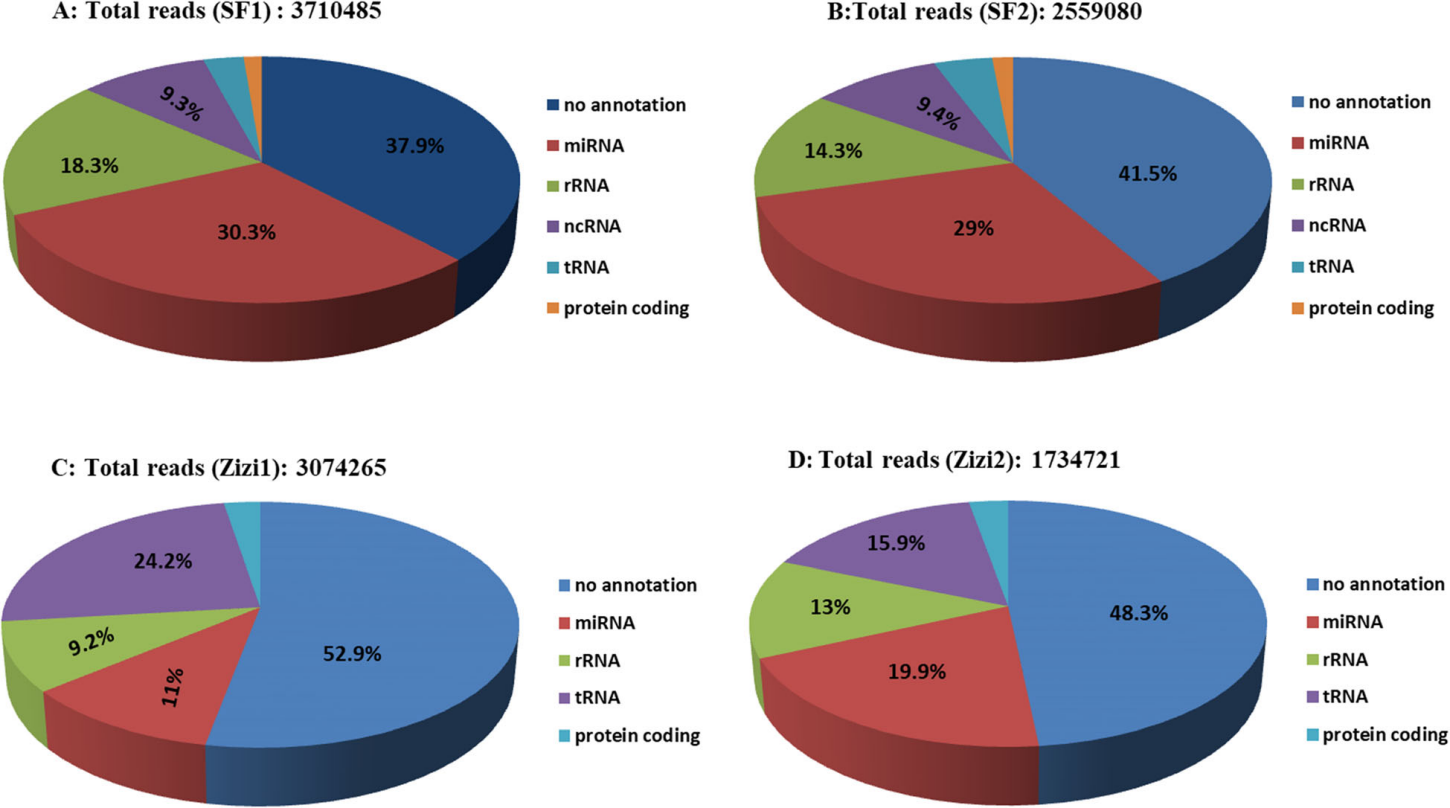
Small RNA annotation overview of sunflower (A and B) and sedr (C and D) pollens. (Since the genome of sedr, was not available, the genome of *Prunus persica* has been used for annotation)

**Fig. 2:**
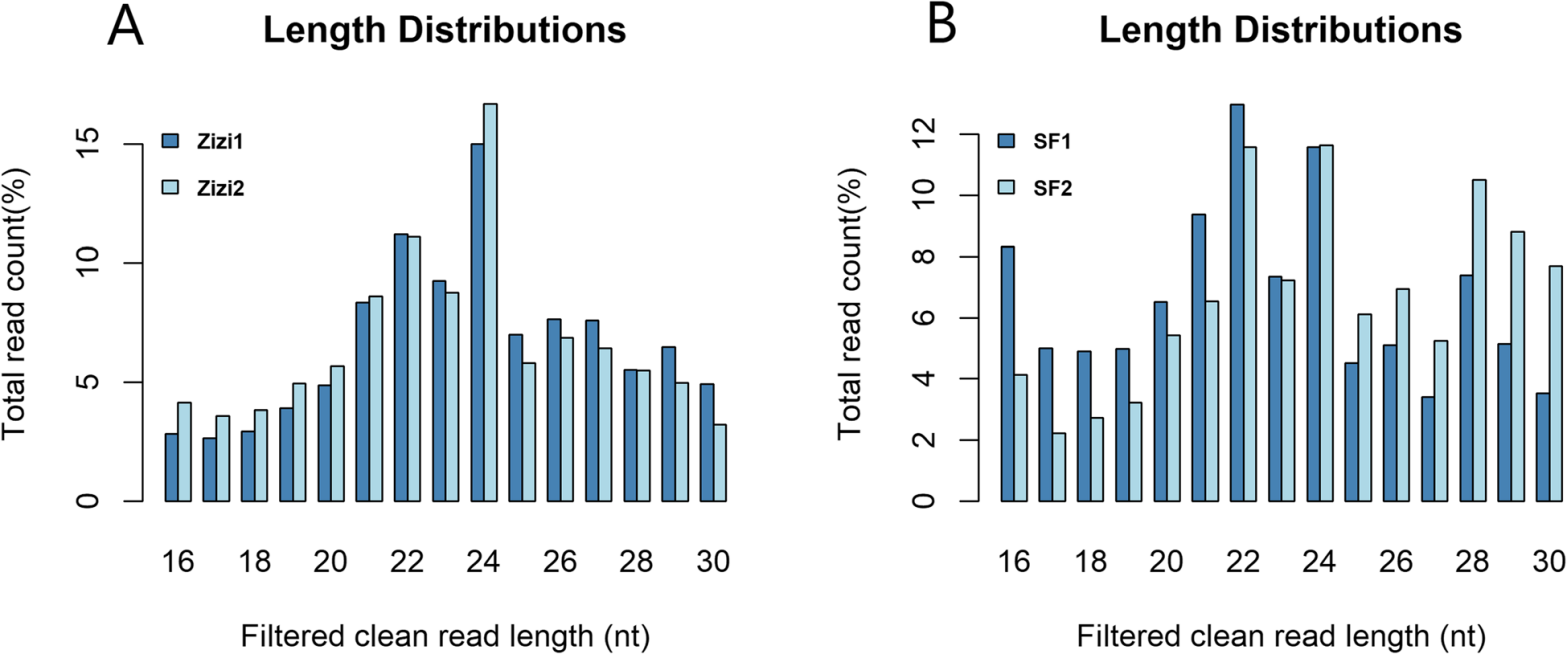
Length distribution of clean reads in *Ziziphus spina-christi* (A) and sunflower (B) pollens. In *Ziziphus* samples 24 nt sRNAs had the largest number, followed by 22 nt sRNAs (Fig. 2A). In sunflower samples 22 nt sRNAs had the highest proportion followed by 24 nt sRNAs (Fig. 2B)

To obtain an overview of the sRNAs repertories in pollen samples, four sRNA libraries were constructed in sunflower and sedr pollen. A total of 3,816,531 (SF1), 2,641,841 (SF2), 3,124,151 (Zizi1), and 1,782,133 (Zizi2) raw reads were created (Table 1). After discarding the low-quality reads, contaminated reads, 3′ and 5′ adaptors and polyA-containing sequences, 3,710,485, 2,559,080, 3,074,265 and 1,734,721 clean reads were obtained in SF1, SF2, Ziz1 and Ziz2, respectively. Subsequently, rRNAs, tRNAs, snRNAs, snoRNAs, low complexity sequences and sequences smaller than 16 nt and longer than 30 nt were filtered, yielding 2,530,144 (SF1), 1,803,175 (SF2), 1,962,507 (Zizi1), and 1,182,018 (Zizi2) clean reads, respectively (Fig. 1; Table 1). The length of pollen sRNAs ranged from 16 to 30 nt, while in sunflower samples 22 nt sRNAs had the highest proportion followed by 24 nt sRNAs (Fig. 2B). In sedr samples 24 nt sRNAs had the largest number, followed by 22 nt sRNAs (Fig. 2A).

**Table 1:** Statistics of small RNA sequences in sunflower and sedar pollen samples

Types	SF1	SF2	Zizi1	Zizi2
Raw reads	3,816,531	2,641,841	3,124,151	1,782,133
Clean reads	3,710,485	2,559,080	3,074,265	1,734,721
rRNA	679,142	365,507	283,842	225,353
tRNA	106,238	106,852	745,454	275,339
snRNA	1001	1439	1297	735
snoRNA	965	591	227	224
Law Complexity reads	1677	883	1526	2301
miRNAs	3733	1567	29,338	41,736
Others	1,120,901	739,670	307,581	303,214
Contaminated reads	133	67	61	90
No annotation	1,405,511	1,061,938	1,625,588	837,068
Reads after the removal of other small RNAs	2,530,144	1,803,175	1,962,507	1,182,018
Aligned reads against known mature plant miRNAs	106,904	29,186	47,478	67,393
Known miRNAs	866	495	423	773

### Known and novel miRNAs in sunflower and Sedr pollen

**Fig. 3:**
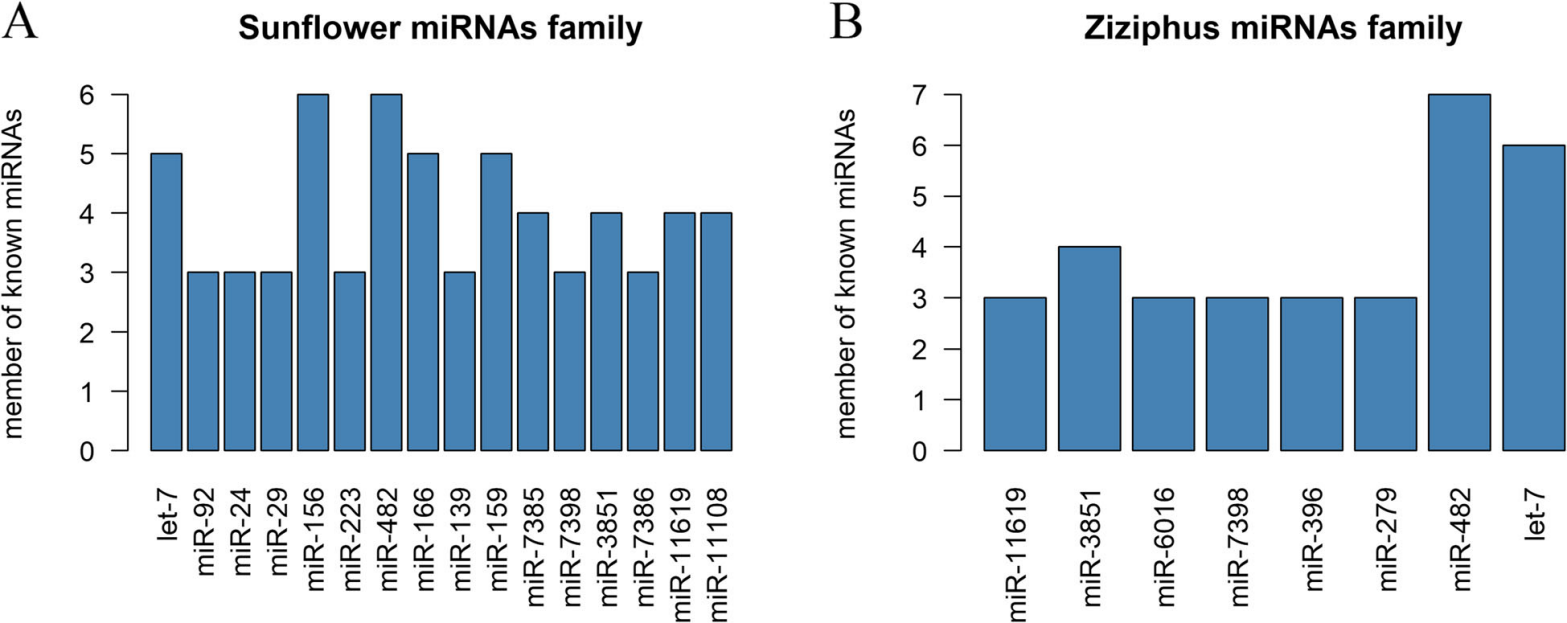
Number of known miRNAs families members in sunflower (A) and *Ziziphus spina-christi* (B) pollens. Since the large number of identified miRNAs families had one or two members in both sunflower and *Ziziphus* pollen, we only presented miRNAs families with more than two members

In order to identify miRNAs in sunflower and sedr pollen samples, the clean sRNA reads were aligned against the miRBase database. In sunflower, a total of 106,904 and 29,186 reads from two samples were mapped to miRBase, which resulted in the identification of 495 shared miRNAs in two samples (Table 1). The identified miRNAs in sunflower were belonged to 409 miRNA families. Among the detected miRNAs, 355 miRNA families consisted of one member; 38 miRNA families consist of two members; seven miRNAs including miR-139, miR-223, miR-24, miR-7386, miR-92, miR-7398 and miR-29 had three members; four miRNAs including miR-11,108, miR-7385, miR-3851 and miR-11,619 had four members, and three miRNAs including miR-166, miR-159 and let-7 had five members. Two largest family, miR-156 and miR-482, had six members (Fig. 3(A), Additional file 1: Table S1).

In sedr pollen samples, a total of 47,478 and 67,393 reads from two samples were mapped to miRBase, which resulted in the identification of 423 shared miRNAs in two samples. In this case, 368 miRNA families were found which 329 miRNA families consisted of one member and 31 miRNA families consist of two miRNAs. Further, miR-396, miR-7398, miR-6016, miR-279 and miR-11,619 had three members, miR-3851 had four members, let-7 had six members and the largest family belongs to miR-482 with seven members (Fig. 3(B), Additional file 2: Table S2). The size of known miRNAs varied from 17 and 28 nt with 22 nt as the peak, followed by 21 nt in both the studied plants.

In addition, novel miRNAs were predicted using miRDeep-P2 software with one mismatch allowed in sunflower pollen. The mapped reads were extended to obtain precursor sequences which were further folded into potential stem-loop structures using Vienna package. The resulted miRNA precursor structures that were represented by less than 10 reads were excluded and a final set of 16 novel miRNAs were predicted (belonging to 10 families and named as Han-novel-miR100 to Han-novel-miR110). The size of novel miRNAs ranged from 20 to 22 nt, while the most of them were 21 nt in length (Additional file 3: Table S3). It has been noted that low free energy is one of the major characteristics of miRNAs [23]. In sedr, unaligned reads to miRBase were subjected to novel miRNAs prediction using Mirnovo software. The list of the identified novel miRNAs, their consensus sequences and coverage features are presented in Additional file 4: Table S4 for sedr plant.

### Diet-derived miRNAs in honey bee Midgut

**Fig. 4:**
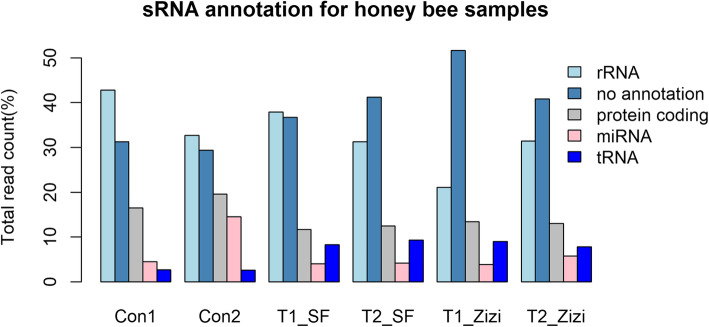
Small RNA annotation of six honey bee samples: T1_SF and T2_SF: honey bees fed with sunflower pollens. T1_Zizi and T2_Zizi: honey bees fed with sedr pollen, and control1 and control2 honey bees fed by sugar syrup

Six sRNA samples were generated from the RNA purified from midgut of honey bee fed with sunflower (two samples) or sedr pollen (two samples). The honey bee fed with sugar syrup was used as the control group (two samples). A total of 2,030,142 (T1-SF), 3,975,267 (T2-SF), 2,237,955 (T1-Zizi), 2,238,200 (T2-Zizi2), 2,660,260 (control1) and 4,251,505 (control2) raw reads were produced (Table 2). After removing rRNAs, tRNAs, snRNAs, snoRNAs and low complexity sequences (Fig. 4; Table 2), the remaining reads were aligned against all insect mature miRNAs from miRBase. Subsequently, unaligned reads were mapped against *Apis mellifera* genome to remove any putative miRNA sequences from honeybee. Finally, 396,963 (T1-SF), 877,506 (T2-SF), 772,823 (T1-Zizi), 597,489 (T2-Zizi), 390,800 (control1) and 466,808 (control2) reads were used for alignment against identified plant miRNAs (Table 2).

**Table 2:** Statistics of small RNA sequences in pollen-treated and control honey bees for detection of diet-derived sunflower and Sedar miRNAs

Types	Control1	Control2	T1_SF	T2_SF	T1_ Zizi	T2_ Zizi
Raw reads	2,660,260	4,251,505	2,030,142	3,975,267	2,237,955	2,238,200
Clean reads	2,558,916	4,093,244	1,696,697	3,449,599	2,077,808	2,172,285
rRNA	1,096,486	1,336,965	642,603	1,079,158	439,323	681,660
tRNA	68,118	108,148	144,150	319,453	187,744	169,690
snRNA	40,226	27,042	16,751	39,478	14,056	15,065
snoRNA	5071	4814	2920	6974	2945	3504
Law complexity reads	4176	10,068	1414	3269	1263	1991
Contaminated reads	938	4651	505	850	623	598
Reads related to *Apis mellifera* miRNAs	111,446	573,125	65,904	140,928	79,284	124,621
Reads related to other insect miRNAs	3097	21,525	1483	2899	1413	1685
No annotation reads	801,690	1,202,178	623,295	1,421,167	1,071,775	887,345
Reads after removal of other small RNAs and all insect miRNAs	801,690	1,202,178	623,295	1,421,167	1,071,775	887,345
Reads mapped against Apis mellifera genome	410,890	735,370	226,332	543,661	298,952	289,856
Reads unmapped against Apis mellifera genome	390,800	466,808	396,963	877,506	772,823	597,489
Aligned reads to SF_miRNAs	56	73	4520	10,632	–	–
Aligned reads to Zizi_miRNAs	45	69	–	–	5670	4100
Count of the detected miRNAs of SF	0	0	11	31	–	–
Count of the detected miRNAs of Zizi	0	0	–	–	15	11

The resulting reads from sunflower- and sedr-treated honey bee samples were aligned against identified sunflower miRNAs (495 known miRNAs and 16 novel miRNAs) and sedr miRNAs (423 known and nine novel miRNAs). As a result, 4520 (T1-SF), 10,632 (T2-SF), 5670 (T1-Zizi) and 4100 (T2-Zizi) reads were aligned to identified miRNAs, which led to detection of 11 (T1-SF), 31 (T2-SF), 15 (T1-Zizi) and 11 (T2-Zizi) miRNAs in the treated groups (Table 2). The miRNAs that were detected in both biological replications of each treatment were kept for further analysis. Interestingly, all the identified miRNAs (11 miRNAs in each group) were detected in both biological replications in both plants. Of these miRNAs, nine miRNAs i.e. miR-148a-3p, miR-26a, miR-21-5p, miR-143, miR-27a, miR-203, let-7 g, miR-126, and miR-30d exist in both two groups (honey bee fed with sunflower and sedr pollen). The miR-101 and miR-103 were only detected in honey bees fed with sunflower, while miR-199b-3p and miR-206 were found in honey bees treated with sedr pollen (Additional file 5: Table S5). The sequences of the control group of honey bees were also aligned against identified sunflower and sedr miRNAs. Fifty-six (control1) and 73 (control2) reads were aligned with sunflower miRNAs and 45 (control1) and 69 (control2) reads were aligned with sedr miRNAs. No common miRNA was found between the control and treated groups.

### Detection of miRNAs using RT-qPCR

**Fig. 5:**
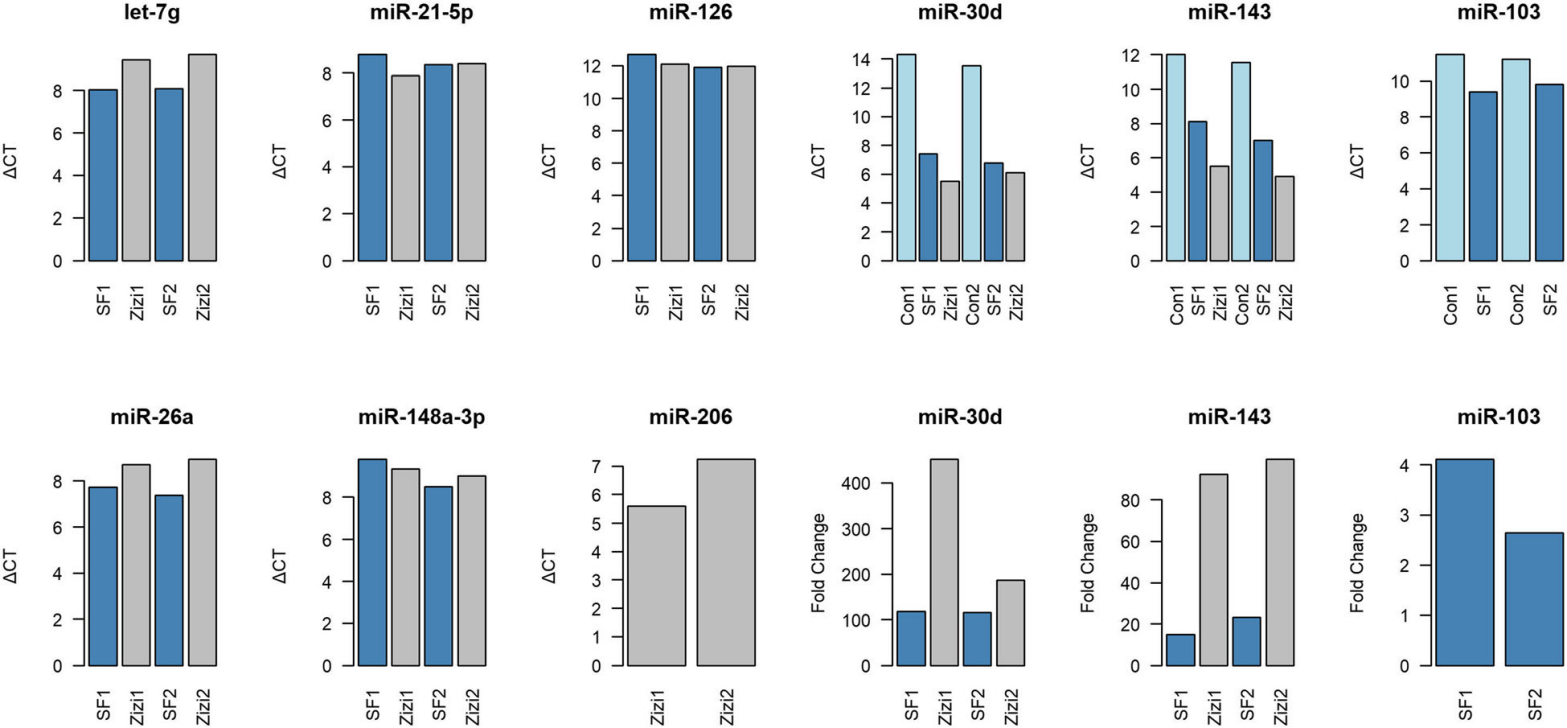
The relative expression of nine diet-derived plant miRNAs in honey bee midgut. Let-7 g, miR-21-5p, miR-126, miR-26a, miR-148a and miR-206 were expressed only in treated groups, however miR-30d, miR-143 and miR-103 were expressed in treated and control groups. Fold change parameter indicated the expression of miR-30d, miR-143 and miR-103 in the treated groups had a significant difference in comparison to the control sample

In order to validate the existence of the identified miRNAs in the midgut, nine miRNAs including miR-30d, miR-143, miR-103, miR-21-5p, let-7 g, miR-26a, miR-126, miR-148a and miR-206 were investigated using RT-qPCR. The expression patterns of six miRNAs including let-7 g, miR-21-5-p, miR-126, miR-26a, miR-148a and miR-206 were consistent between RT-qPCR and RNA-Seq (Fig. 5). However, the expression patterns of three miRNAs including miR-30d, miR-143 and miR-103 were not consistent between RT-qPCR and RNA-seq. (Fig. 5). Out of seven shared miRNAs between the two treated groups, there was no amplification in control samples in five miRNAs i.e. miR-21-5p, let-7 g, miR-26a, miR-126, and miR-148a-3p, while in the treated samples all the five miRNAs were expressed. Two miRNAs including let-7 g and miR-26a were highly expressed in sunflower-treated honey bees than sedr treated groups. (Student t-test, *p* < 0.05). However, the expressions of three miRNAs including miR-21-5p, miR-126 and miR-148a-3p were similar between sunflower and sedr treated groups. In the case of miR-143 and miR-30d, extension was observed in the control group in addition to the treated groups. On the other hand, miR-206 was expressed in sedr treatment but not in the control group, while miR-103 was expressed in both the control and sunflower treatment. For the three miRNAs that were expressed in control group even more than the treated groups fold change parameter was calculated. The expression of miR-30d, miR-143 and miR-103 in the treated groups showed a significant difference in comparison to the control sample. The lower expression of the three miRNAs in the control group might be due to the differences in feeding (lower feeding) of the honey bees inside the colony.

### Target prediction and potential function of individual miRNAs

In total, 121 target genes were predicted for the 11 diet-derived plant miRNAs. Only one target was predicted for miR-203 and miR-101, while 21, 11, 8, 7, 7, 6, 4 and 2 target genes were predicted for miR-103, let-7 g, miR-143, miR-199b, miR-126, miR-27a, miR-26 and miR-30d, respectively. The miR-206 had the highest number of targets with 53 predicted genes (Additional file 6: Table S6) and no taget gene was detected for miR-148a and miR-21-5p.

**Fig. 6:**
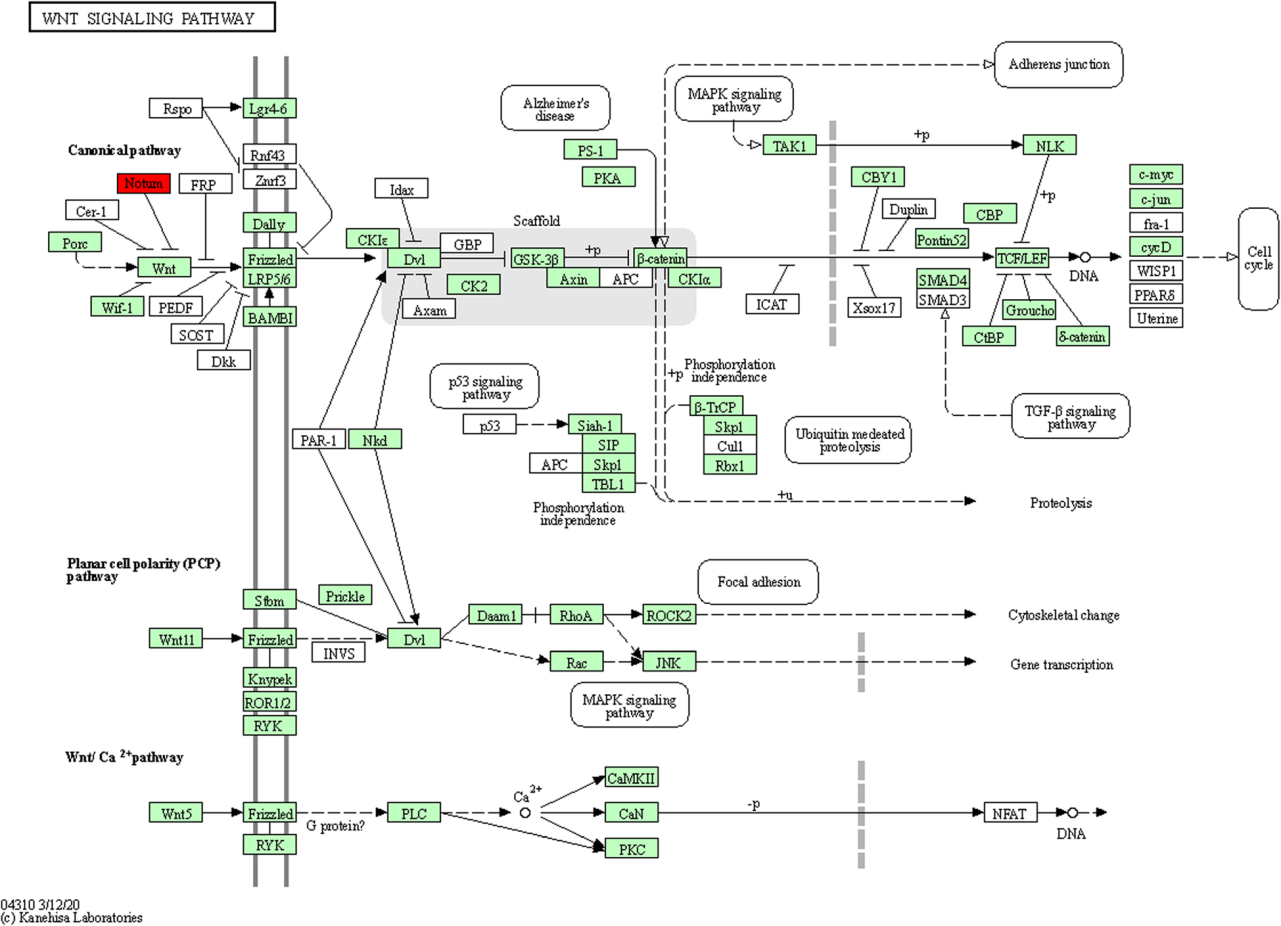
KEGG enrichment analysis of the wnt signaling pathway in honeybee by KOBAS 3.0 online tool (http://kobas.cbi.pku.edu.cn) and [24]. Regulation of the *wnt* signaling pathway in honeybee midgut by exogenous let-7 g via targeting genes: GB54903 and GB49269. Red box represent genes identified in our study. The genes in green background represent the species specific genes (*Apis mellifera*)

**Fig. 7:**
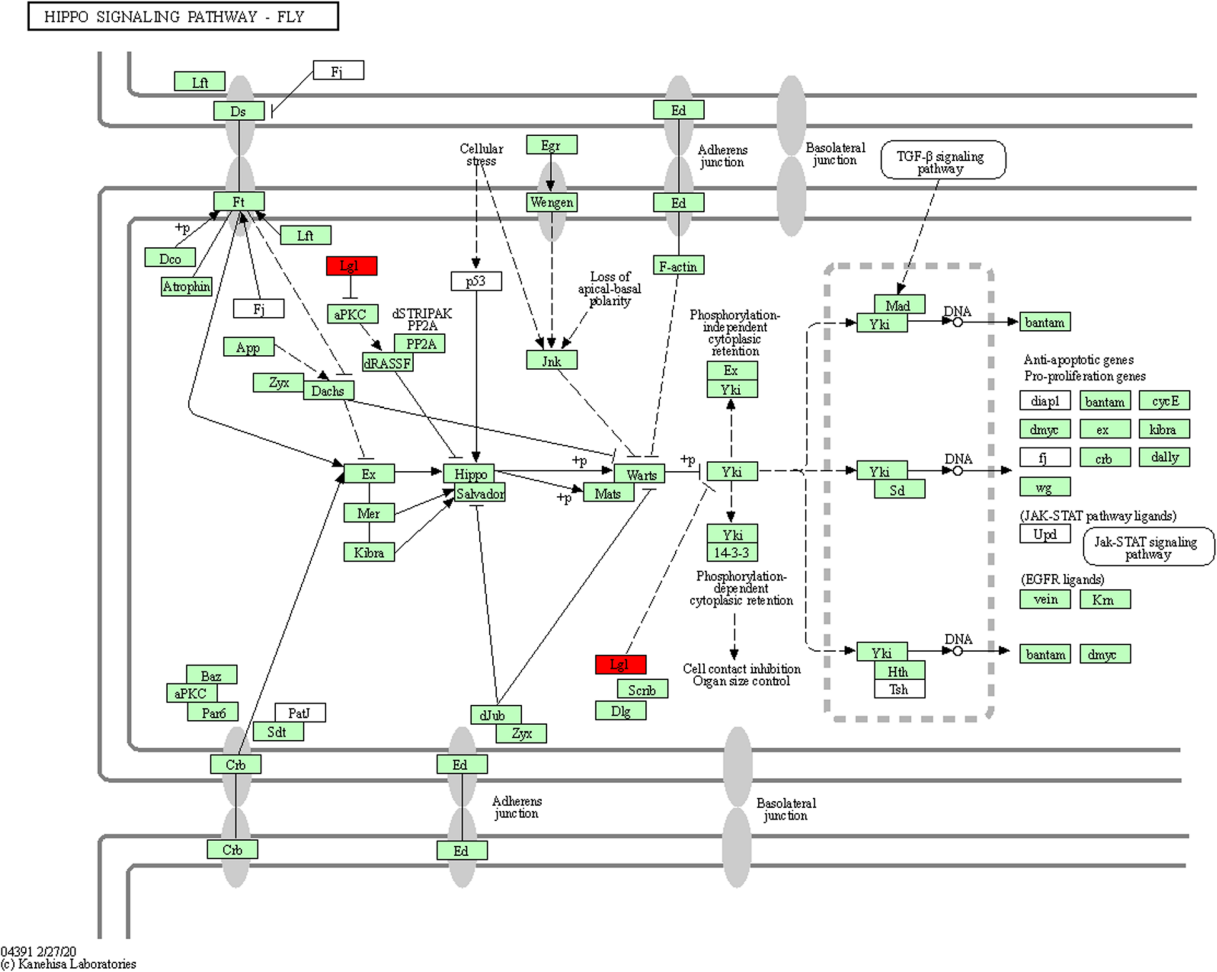
KEGG enrichment analysis of the hippo-signaling pathway-fly in honeybee by KOBAS 3.0 online tool (http://kobas.cbi.pku.edu.cn) and [24]. Regulation of the hippo-signaling pathway-fly in honeybee midgut by exogenous let-7 g via targeting gene: GB55164. Red box represent genes identified in our study. The genes in green background represent the species specific genes (*Apis mellifera*)

To shed a light on the potential function of the target genes of transmitted plant miRNAs, KEGG pathway analysis was performed. Totally, 25 pathways related to eight diet-derived miRNAs were significantly enriched (Table 3). The exogenous let-7 g targets were significantly enriched in Wnt signaling pathway such as Palmitoleoyl-Protein Carboxylesterase NOTUM (LOC107963993) and Palmitoleoyl-Protein Carboxylesterase NOTUM-like (LOC552168) (Fig. 6). *Wnt* signaling is one of the most crucial morphogens for development and associated with the establishment and maintenance of synaptic structural and neuronal function during the maturation of the central nervous system [25]. The other important target genes of let-7 are Lethal (2) giant larvae protein homolog 1 (LOC409348), which is involved in hippo signaling pathway (Fig. 7). Hippo signaling pathway is an evolutionarily conserved signaling pathway that plays an important role in the control of organ growth and development [26]. Interestingly, previous study in honey bee revealed that development of morphological differences among honey bee castes require a complex fine-tuning of hippo signaling pathway by miRNAs and DNA methylation [17]. Pyruvate carboxylase (LOC412876) is the other predicted target gene by exogenous let-7. This gene is involved in pyruvate metabolism, citrate cycle (TCA cycle), biosynthesis of amino acids and carbon metabolism. Also, phosphatidylinositol 4-kinase type 2 (GB50211) was predicted to be targeted by exogenous let-7. This gene plays important roles in Inositol phosphate metabolism and phosphatidylinositol signaling pathways. N-Glycan biosynthesis pathway was identified to be enriched by the target genes of miR-26a. The pathways associated with protein biosynthesis and lipid metabolism as well as glycan biosynthesis and metabolism [N-glycan biosynthesis and Glycosylphosphatidylinositol (GPI)-anchor biosynthesis] are important for active nurse bees to synthesize the major protein components of royal jelly and secrete the lipid-rich components to the brood [27]. Hepatocyte growth factor-regulated tyrosine kinase substrate (Gene Hrs) was predicted to be targeted by exogenous miR-27a and is involved in endocytosis and phagosome pathways. Hrs plays a key role in the invagination of the endosome membrane and the formation of multi-vesicular bodies (MVBs). Hrs appeared to be essential during gastrulation and regulated early embryonic signaling pathways. Furthermore, Hrs may inhibit tyrosine kinase receptor signaling by promoting tyrosine phosphorylate degradation of the active receptor, potentially through sorting activated receptors into MVBs. Seven genes were identified as targets of miR-126 and were significantly enriched in seven different pathways including: retinol metabolism, tyrosine metabolism, drug metabolism-cytochrome P450, metabolism of xenbiotics by cytochrome P450, fatty acid degradation, glycolysis gluconeogenesis and carbon metabolism pathways. One of the target genes is Fdh (S-(hydroxymethyl) glutathione dehydrogenase) with catalytic activity. Our results indicated that miR-103 is capable of targeting collagen alpha-1(IV) chain (LOC408552), which is involved in ECM-receptor interaction pathway in the honey bee. Previously, it has been shown that the ECM-receptor interaction pathway is related to high royal jelly production in the honey bee [28]. One of the most important potential target genes of miR-199 was Signal sequence receptor subunit gamma (LOC552295), which acts as TRAP proteins. This gene binds calcium to the endoplasmic reticulum (ER) membrane and thereby regulates the retention of ER resident proteins. Interestingly, it has been reported that divergent forms of endoplasmic reticulum stress generate a strong unfolded protein response in honey bees [29]. In the present study, bioinformatics analysis indicated exogenous miR-206 had the highest number of targets with 53 predicted genes. Enrichment analyses of miR-206 target genes indicated target genes participated in 21 different pathways while none of the identified pathways was significant (Additional file 7: Table S7). All these findings will extend our understanding of the molecular interactions between honey bee and flowering host plants.

**Table 3:** Significant KEGG pathways in the target genes of the detected diet-derived plant miRNAs

miRNAs	PATHWAY	Gene Name	*P*-Value	Corrected-*P*-Value
Let-7 g	ame04310:Wnt signaling pathway	GB54903, GB49269	1.70e-3	1.53e-2
	ame00620:Pyruvate metabolism	GB40280	2.95e-2	5.94e-2
	ame00020: Citrate cycle (TCA cycle)	GB40280	3.22e-2	5.94e-2
	ame00562: Inositol phosphate metabolism	GB50211	4.27e-2	5.94e-2
	ame04070:Phosphatidylinositol signaling	GB50211	4.62e-2	5.94e-2
	ame04391:Hippo signaling pathway-fly	GB55164	4.62e-2	5.94e-2
	ame01230:Biosynthesis of amino acids	GB40280	4.62e-2	5.94e-2
	ame01200: Carbon metabolism	GB40280	8.030e-2	9.04e-2
miR-126	ame00830:Retinol metabolism	GB53086	3.95e-3	1.18e-2
	ame00350: Tyrosine metabolism	GB53086	4.94e-3	1.18e-2
	ame00982:Drug metabolism-cytochrome P450	GB53086	5.27e-3	1.18–2
	ame00980:Metabolism of xenobiotics by cytochrome P450	GB53086	5.92e-3	1.18e-2
	ame00071:Fatty acid degradation	GB53086	8.22e-3	1.32e-2
	ame00010: Glycolysis Gluconeogenesis	GB53086	1.41e-2	1.88e-2
	ame01200: Carbon metabolism	GB53086	3.00e-2	3.43e-2
miR-143	ame00970:Aminoacyl-tRNA biosynthesis	GB40563	1.68e-2	1.68e-2
miR-103	ame04512:ECM-receptor interaction	GB45968	2.77e-2	3.99e-2
	ame04933:AGE-RAGE signaling pathway in complications	GB45968	3.99e-2	3.99e-2
miR-199	ame03040-Spliceosome	GB52855	3.42e-2	3.71e-2
	ame04141:Protein processing in endoplasmic reticulum	GB41586	3.71e-2	3.71e-2
miR-26a	ame00510:N-Glycan biosynthesis	GB54846	5.77e-3	5.77e-3
miR-203	ame00230:Purin metabolism	GB44602	6.19e-3	1.24e-2
	ame01100:Metabolic pathways	GB44602	6.70e-2	6.70e-2
miR-27a	ame04145:Phagosome	GB54140	9.87e-3	1.82e-2
	ame04144:Endocytosis	GB54140	1.82e-2	1.82e-2

## Discussion

Recent study supports evidence which movement of small RNA molecules (siRNA and miRNA) between species may play a key role for the dissemination of gene-silencing signals and the promotion of cross-talk between different organisms [30]. Taken together, the mobility of small RNAs from one species to another has been broadly supported from bacteria to nematodes [31], from fungal pathogens to plants [32], from plants to pathogenic and symbiotic microbes [33–36], from plants to nematodes [37] and from plants to vertebrates [12, 15].

Diet-derived transmission of miRNAs from host plant into the midgut of honey bees was evaluated under environmentally controlled conditions. It has noted that horizontal transmission of miRNAs to recipient honey bees is negligible [22]. They investigated transmission of miR-156a, the highly conserved plant miRNAs in pollen in different tissues of honey bees in different life stages of the insect. Though they have observed robust increase of plant miRNAs in the midgut tissue after pollen ingestion, they found no evidence of biologically relevant function for delivery of these molecules to proximal or distal tissues of recipient honey bees. However, our work presented evidence that a number of plant miRNAs were successfully transmitted to the midgut of honey bee. We showed that the transmission of diet-derived plant miRNAs into adult honey bee midgut tissue under controlled pollen feeding conditions. This is the first comprehensive study confirming the transmission of miRNAs from the host plant into honey bees.

The let-7 miRNA was one of the first miRNAs discovered in the nematode *Caenorhabditis elegans* [38], which is involved in various developmental processes as shown in *Bombyx mori* and *Drosophila melanogaster* [39, 40]. Previous studies in honey bee demonstrated that Let-7 is one of the few consistent miRNA markers associated with the behavioral shift of worker bees from nurses to foragers [41–43]. Recently bioinformatics analysis has suggested that Wnt signaling pathway play a critical role in honey bee behavioural maturation [44]. To determine the relationship between honey bee nursing performance and neurobiological activities, phosphoproteom investigation in nurse honey bee brains revealed that phosphatidylinositol signaling pathway, inositol phosphate metabolism and Wnt signaling pathways are required for brain maturation, development, signal transduction and the olfactory learning processes during the early stages of worker life to enhance their nursing task performance [45]. This finding may reinforce the results of our study that enriched Wnt signaling pathway, phosphatidylinositol signaling pathway and inositol phosphate metabolism in the exogenous let-7 genes target may be involved in the modulation of honey bee behavior by regulating the neuronal function of the honey bee brain. However more comprehensive studies are needed to prove the the role of exogenous let-7 target genes in honey bee nursing task performance. miR-143 is known as a multi-functional miRNA. Predicted target genes by exogenous miR-143 were significantly enriched in aminoacyl-tRNA biosynthesis pathway. Previously, the role of aminoacy-tRNA biosynthesis pathway in processing of materials of nascent peptides for protein synthesis in honey bee (48 h embryos) has been indicated [46]. However, further works are required to illustrate the role of exogenous miR-143 target genes for protein synthesis in honeybee.

Phagocytosis is related to cellular immune pathway in insects [47]. Previously over-representation of phagosome formation pathway has been observed in nurse honey bees when compared to forager bees [27]. Over-representation of this pathway in nurse honey bees can be led to enhance the immune activity in nurses, which is necessary to brood care. This finding is consistent with our result that suggested target genes by exogenous miR-27 were significantly enriched in phagosome pathway in nurse honey bee. However, further investigations are required to elucidate the roles of exogenous miR-27 target genes in immune pathway. The other enriched pathway by exogenous miR-27 was endocytosis, which may be involved in honey bee antiviral defense [48]. miR-206, is one of the interesting miRNAs characterized in our study which has a significant effect on the dynamic mechanism of the mammalian circadian rhythms occurring with a periodicity close to 24 h in almost all living organisms from the cyanobacteria to plants, insects and mammals [49]. Although it has not yet been mentioned the role of miR-206 in controlling the circadian clock of insects, the results of present study may be a helpful step to evaluate this idea.

## Conclusions

We demonstrate that plant miRNAs could be transmitted into the midgut of honey bee upon the insect feeding on pollen. Our findings also revealed the lack of obvious differences between miRNAs originated from sunflower and sedr pollen, although some unique miRNAs for each of the host plants were detected in the insect body. We also determined the target genes for all the miRNAs with plant origin in the midgut of honey bee. KEGG analyses proposed the involvement of the target genes in different pathways that may help establish the conditions for a compatible interaction. Our results revealed many candidate genes which could be involved in the transmission and compatibility of plant miRNAs into the midgut of honey bee that may be potentially useful for engineering molecular aspects of pollinator insects in the twenty-first century’s agriculture. Finally, based on the findings of present study, it is suggested that the probability of plant miRNAs being transferred from nurse bees to larvae during feeding be explored further in the future.

## Methods

### Honey bee and pollen collection

A healthy honey bee (*Apis mellifera meda*) colony with no symptoms of common bacterial, fungal and viral diseases was settled next to a sunflower farm in Shal County of Qazvin province (located in central of Iran) in September 2018, when %80 > of the plants were in flowering stage. A similar colony was settled next to a wide area of sedr trees in Jiroft County of Kerman province (located in south of Iran), during their flowering stage in October 2018. Pollen was collected from corbiculae of returning forager bees using pollen trap device and immediately transferred into a fluid nitrogen tank.

### Feeding treatments

For feeding experiment, nurse bees that pushed their heads in older larval cells for at least 3 s, were collected using mouth aspirator. In brief, about a hundred young nurse bees, ranging in age from three to 6 days, were transferred to each cage. Bees from two replicate of each treatment were collected from the same colony. The outer dimensions of the cages were 15 × 11 × 15 cm. Except for the front portion; the cages were entirely made of wood. The front of the cages was made of 8-mesh screen (screen cell opening were 1.5 mm wide). The top of the cages had two holes where feeding tubes could be inserted. Within the cages for resting bees, a small empty comb was attached. The bees were placed inside the cages by sliding one side of the cages. The cages were placed inside the hives to maintain natural conditions including Queen Mandibular Phermone (QMP), humidity, as well as temperature, and metal mesh nets with 1 mm holes were used to separate the hive from the cages. All the bee groups were fed with a diet of sugar syrup for 48 h, starved for 3 h to encourage feeding, and then two cages were fed with 30% sucrose + sunflower pollen at a concentration of 50 mg/ml, two cages were fed with 30% sucrose + sedr pollen and the other two cages were fed with sucrose syrup without pollen as a control group for 24 h. After a cold anesthesia, honey bee midgut tissues were aseptically removed from abdomens for further analyses. The food bolus was removed from the midgut of treatment group by sliding out the peritrophic matrix as 1 or 2 discrete sacs with a sterile scalplel, along with its contents. All the collected midgut samples as well as the pollen samples used for feeding, were resolved in Trizol (Invitrogen) and stored at − 80 °C to be used for total RNA extraction.

### RNA extraction and sequencing

Total RNA from sunflower, sedr pollens and honey bee midgut tissues including those fed by sunflower and sedr pollen as well as the control bees fed by sugar syrup (two replicates per treatment, named: SF1, SF2, Zizi1, Zizi2, T1-SF, T2-SF, T1-Zizi, T2-Zizi, Con1 and Con2 respectively) was extracted using miRNeasy mini Kit (Qiagen, Valencia, CA, USA) with a phenol-chloroform column-based extraction method as previously described by [22]. The quality and contamination of the RNAs were monitored on 1% agarose gel. Furthermore, RNA purity was checked using the NanoPhotometer® spectrophotometer (IMPLEN, CA, USA), while the concentration was measured using Qubit® RNA Assay Kit in Qubit® 2.0 Flurometer (Life Technologies, CA, USA). RNA integrity was also assessed using the RNA Nano 6000 Assay Kit of the Agilent Bioanalyzer 2100 system (Agilent Technologies, CA, USA). The cDNA libraries were generated using NEBNext® Multiplex Small RNA Library Prep Set for Illumina® (NEB, USA) and were clustered using TruSeq SR Cluster Kit v3-cBot-HS according to the manufacturer’s instructions. They then were subjected to sequencing on an Illumina Hiseq 2500/2000 platform and 50 bp single-end reads at Novogene (HK) Company- Beijing, China (https://en.novogene.com/). The sequencing results were deposited in the NCBI SRA database under the project PRJNA695090.

### MiRNA identification

Briefly, clean reads were obtained after discarding adapters, poly-N sequences, and low quality reads smaller than 16 nt or longer than 30 nt. The clean Reads were aligned to a collection of Silva, GtRNAdb, Rfam, and Repbase databases to filter ribosomal RNAs (rRNA), transfer RNAs (tRNA), small nuclear RNAs (snRNA), small nucleolar RNAs (snoRNA), repeat sequences and other ncRNAs using Bowtie tool (v1.1.2) [50]. Since the genome of sedr was not available, the genome of *Prunus persica* (accession number: GCA_000346465) was used for annotation. The resulted clean reads were used to detect known miRNAs, in sunflower and sedr pollens, through comparing them against plant mature miRNAs from miRBase Release 22 (http://www.mirbase.org) using Bowtie alignment tool (v1.1.2) with one mismatch. Then, unaligned unique reads were applied to identify novel miRNAs in in sunflower and sedr pollens separately. RNAFold tools [51] were used to predict the secondary structure of all the novel miRNAs. miRDeep-P2 (v1.1.3) software [52], with one mismatch allowed was used to predict the novel miRNAs in the unaligned unique reads of sunflower samples. The novel miRNAs prediction in miRDeep-P2 revolves around the chromosome id, strand direction, mature miRNA sequence, miRNA location, precursor sequence, precursor location, hairpin structure and minimum free energy. Reference genome of sedr had not been sequenced yet. Hence, to predict the novel miRNAs in sedr samples Mirnovo software with default parameters, was used, which is able to identify miRNAs directly from small RNA-seq data with or without reference genome [53].

On the other hand, diet-derived miRNAs in honey bee midgut samples were detected using Bowtie alignment tool with one mismatch allowed. To do this end, clean reads of the honey bee samples were mapped against mature insect miRNAs from miRBase to remove known and conserved insect miRNAs. Unmapped reads to insect miRNAs were aligned against *Apis mellifera* genome (accession number: GCA_000002195.1) to exclude the reads related to potential novel honey bee miRNAs, which have not been reported yet. Finally, the remaining reads were aligned against the identified sunflower and sedr miRNAs (obtained from the previous steps) to detect diet-derived miRNAs in the midgut tissues.

### miRNA target prediction

To predict the potential targets of the miRNAs of interest, 3’UTR of the annotated genes of honey bee were searched. For genes lacking a predicted 3′ UTR, 500 bp downstream of the stop codon was selected as described by [17]. To minimize the false positive results, three different target prediction tools including PITA, Miranda and RNAHybrid were applied with a maximum free energy of − 15 kcal/mol. Those targets that were predicted by two out of the three tools were selected as potential targets of each gene. Kyoto Encyclopedia of Genes and Genome (KEGG) pathway analysis for the target genes of each miRNA was performed using KOBAS 3.0 online tool (http://kobas.cbi.pku.edu.cn) and [24]. In addition, corrected *P*-value< 0.1 was set as the cut-off criterion.

### RT-qPCR

Out of 13 detected plant-derived miRNAs in honey bee midgut, nine miRNAs were randomly selected for further validation using RT-qPCR. The miDETECT A Track Uni-Reverse primers and miDETECT A track miRNA forward primers (specific primers) were used for amplification of the target miRNAs in RT-qPCR (Ribobio CO., Ltd.Guangzhou, China). Poly(A) tailing, reverse transcription and qPCR were performed successively using the miDETECT A Track miRNA qRT-PCR starter kit (Ribobio Co., Ltd). The RT-qPCR reactions were performed using SYBR green mix in 96 well-plates with two biological replications and three technical replications using Actin (Forward Primer: TGCCAACACTGTCCTTTCTG, Reverse Primer: AGAATTGACCCACCAATCCA) as the endogenous control. The PCR conditions used were as follow: 10 min at 95 °C, 40 cycles of 2 s at 95 °C, 20 s at 60 °C, 10 s at 70 °C and one cycle for melting curve generation. The relative expression level of miRNAs was calculated using CT and ΔCT method [54].

## Supplementary Information

**Additional file 1 Table S1**. Identification of known miRNAs in sunflower pollen. The clean sRNA reads were aligned against the miRBase database which resulted in the identification of 495 shared miRNAs in two samples.

**Additional file 2 Table S2**. Identification of known miRNAs in sedr pollen. The clean sRNA reads were aligned against the miRBase database which resulted in the identification of 423 shared miRNAs in two samples.

**Additional file 3 Table S3**. Identification of novel miRNAs in sunflower pollen. 16 novel miRNAs were predicted in sunflower pollen using miRDeep-P2 software. The predicted miRNAs were belong to 10 families and named as Han-novel-miR100 to Han-novel-miR110.

**Additional file 4 Table S4**. Identification of novel miRNAs in sedr pollen. Nine novel miRNAs were predicted in sedr pollen using Mirnovo software. miRNAs sequences and theirs coverage features are presented in this table.

**Additional file 5 Table S5**. Detection of sunflower and sedr miRNAs in honey bee midgut. The miRNAs that were detected in both biological replications of each treatment were kept for further analysis. Interestingly, all the identified miRNAs (11 miRNAs in each group) were detected in both biological replications in both plants. Of these miRNAs, nine miRNAs i.e. miR-148a-3p, miR-26a, miR-21-5p, miR-143, miR-27a, miR-203, let-7 g, miR-126, and miR-30d exist in both two groups (honey bee fed with sunflower and sedr pollen). The miR-101 and miR-103 were only detected in honey bees fed with sunflower, while miR-199b-3p and miR-206 were found in honey bees treated with sedr pollen.

**Additional file 6 Table S6**. Target prediction of 11 diet-derived miRNAs in honey bee via PITA, Miranda and RNAHybrid softwares. Those targets that were predicted by two out of the three tools were selected as potential targets of each gene. In total, 121 target genes were predicted for 11 diet derived miRNAs.

**Additional file 7 Table S7**. KEGG pathway analysis of miR-206 target genes. This miRNA had the highest number of targets with 53 predicted genes. Enrichment analyses of miR-206 target genes indicated target genes participated in 21 different pathways while none of the identified pathways was significant.

## Data Availability

The relevant data and additional information are available in the supplementary files and also from the corresponding author upon reasonable request. Furthermore, the raw small RNA-Seq data were deposited in the NCBI Sequence Read Archive under BioProject number PRJNA695090: https://www.ncbi.nlm.nih.gov/sra/PRJNA695090.
